# Enabling pupils to flourish: six evidence-based principles of whole-school wellbeing promotion

**DOI:** 10.3389/fpubh.2024.1335861

**Published:** 2024-08-29

**Authors:** Rowan Edwards, Jennifer Byrne, Marcus Grace

**Affiliations:** School of Education, University of Southampton, Southampton, United Kingdom

**Keywords:** health and wellbeing, whole-school, health promotion, flourish, primary school, social and emotional learning

## Abstract

Schools have become increasingly important as health promotion settings, seeking to improve pupils’ health and wellbeing through adopting a whole-school approach. A strong evidence-base highlights that focusing on the social, emotional and psychological aspects of pupils’ wellbeing enables them to flourish, enjoy life and be better equipped to overcome challenges. However, it is acknowledged that further evidence is required regarding: (1) what happens in primary schools, (2) the impact of the English education system, (3) complexity and context, and (4) capturing children’s voices. This article, therefore, addresses these gaps by asking the question: **How do schools use whole-school wellbeing promotion to enable pupils to flourish?** Taking an exploratory approach the study used a three-phase, mixed methods design to address the research problem by undertaking a systematic literature review, a secondary data analysis and a case study to capture multiple stakeholder voices including pupils. As appropriate for this research design, the findings from each phase were integrated into an overarching analysis which is presented in this article. Six broad principles formed consistent threads across the findings: (1) enabling children to flourish, (2) integrating wellbeing with key school goals, (3) promoting wellbeing and building capital, (4) building on virtuous cycles, (5) managing complexity and context, and (6) evaluating wellbeing promotion through listening to different voices. As well as presenting new knowledge addressing the identified research gaps, this study has demonstrated that schools can avoid ‘*reinventing the wheel*’ by adopting existing practices and resources and adapting them to their own setting. It is, therefore, hoped the six evidence-based principles of this study are equally transferable to schools within the English education system and more broadly. In addition, the paper highlights recognized challenges to staffing and resourcing and raises the question over whether schools receive sufficient funding to deliver the whole-school initiatives that government recommends. This article provides readers with an exploration of what has been achieved in schools and it is outside its scope to address specific issues about funding and other practical logistics for implementing whole-school wellbeing promotion, therefore further research is recommended.

## 1 Introduction

In the last three decades schools have become increasingly important settings to promote pupils’ health and wellbeing (1–3). Whilst the benefit of physical health is firmly established and reflected in schools’ curricula, more recently there has been a growing emphasis on schools’ role in promoting the social, emotional and psychological aspects of wellbeing. High levels of wellbeing are associated with pupils flourishing. This paper uses Seligman’s (4) definition of flourishing based on five pillars: positive emotion, engagement, relationships, meaning, and accomplishment. It is recognized that where children flourish they enjoy life more and cope better with adversity (5, 6). As a result, countries have translated these global guidelines into national policies, including the Every Student Succeeds Act in the United States (7), the provision for student health and wellbeing in Australia (8) and mandatory mental health and relationships education in England, requiring all schools to promote skills and knowledge associated with social and emotional wellbeing (1). In the English education system, education staff are involved in promoting wellbeing within their schools.

Simultaneously there is increasing awareness that children are challenged by aspects of their social, cultural, educational and economic circumstances. Increasing income gaps, poorer health amongst children from disadvantaged backgrounds and the under-performance of pupils from certain groups negatively impacts on wellbeing (9–11). Furthermore, growing exposure to technology, increased school testing and complex family situations require children to develop adaptive skills and resilience, which research has shown is enhanced by school-based initiatives (12–14). As well, in England, in 2020 and 2021 pupils were severely impacted by two periods of school closure due to the COVID-19 pandemic, also recognized as detrimental to wellbeing (15).

Nutbeam (16) identifies the roles schools play in developing pupils’ wellbeing skills, promoting health literacy and providing a supportive culture, and further evidence shows that a ‘whole-school’ approach is considered most appropriate (17, 18). A whole-school approach promotes the wellbeing of the school community, through a taught curriculum in combination with a ‘*health enhancing social and physical environment*’ (19) (p. 26). This reflects Dooris et al.’s (20) argument that the setting itself influences pupils’ outcomes. Significant evidence demonstrates that where such activities are undertaken, pupils experience higher levels of wellbeing alongside improved academic performance (17). Literature also highlights that health promoting strategies typically adopt salutogenic principles (21). Antonovsky’s (21) (p. 14) concept of salutogenesis suggests that people lie on a continuum of ‘*ease*’ and ‘*dis-ease*.’ A salutogenic approach seeks to understand the factors which allow an individual to move toward ‘*ease*,’ through promoting higher levels of wellbeing (22).

Despite this solid evidence base, Goldberg (23) suggests that initiatives may fall short, focusing on delivering curriculum content to pupils and failing to consider other aspects of children’s education. Other empirical research appears to reflect this argument, with two large-scale reviews of whole-school wellbeing promotion demonstrating that interventions are often classroom-based and do not seek to make changes to the school’s ethos or organization (17, 18). Perhaps, as Rutter et al. (24) suggest, this arises from school leadership seeing health promotion as a cause-and-effect process, whereby they seek to fix a problem when it arises. Instead, it appears that whole-school wellbeing promotion is more sustainable where it is seen as a process of continual change, affecting all structures and processes (18, 25).

Additionally, evidence emphasizes the importance of recognizing complexity in health promotion. Where complexity is understood, health promotion benefits from being tailored to the local environment and population (26, 27). In school settings, a series of recent systematic reviews and meta-analyses have demonstrated that where wellbeing promotion acknowledges context and complexity this results in more relevant and sustained outcomes (18, 25, 28). Nastasi and Schensul (26) and Hill et al. (29) strengthen this argument by suggesting that a ‘*one size fits all*’ approach may not only be short lived but may fail to reach all groups within a population. Leading academics also argue that it is not only the environment that influences children’s wellbeing, but also a child’s contextual embeddedness from which they cannot be separated (30–33). Rees, Goswami and Bradshaw (34) outlined a range of facilitators and barriers to wellbeing amongst children living in the European Union, identifying that:

*Children interact with their environment and thus play an active role in creating their well-being by balancing the different factors, developing and making use of resources and responding to stress* (p. 136).

Whilst existing literature provides a strong evidence base to inform whole-school wellbeing promotion, some researchers identify remaining knowledge gaps. Firstly, whole-school wellbeing promotion literature has focused primarily on young people in secondary level education (18, 28, 35, 36). Despite many widely-cited reviews, it appears that only two reviews have solely focused on the primary school setting (28, 37). Whilst Adi et al.’s (37) systematic review on mental health promotion in primary schools has been influential within UK education, it is now 15 years old. Fenwick-Smith, Dahlberg and Thompson’s (28) systematic review is more recent, yet its inclusion of seven programs across 11 studies demonstrates the limited scope of high-quality evidence at primary-school level. Secondly, to date, most publications relate to practices in high income countries, with literature predominantly published in the US and Australia, and to a lesser extent Europe (17, 25, 36). Durlak et al. (17) (p. 420) argue maximum benefits exist where schools adopt ‘*programs that fit best with local settings*.’ This is echoed by Weare and Nind (18) (p. 66) who call for schools to adopt initiatives that ‘*fit their context and can be easily implemented*.’ Therefore, this study sought to focus on the English education system, that is currently underrepresented in the literature. Thirdly, alongside a call for additional evidence in whole-school wellbeing promotion, academics highlight that this should include consideration of the impact of contextual factors. In their review, O’Reilly et al. (36) call for greater recognition of environment, calling for future research amongst different populations and contexts. This is echoed by Pawson and Tilley’s (38) (p. 85) requirement for closer focus on the characteristics of the environment to ‘*determine what works for whom and under what circumstances*.’ Lastly, current discourse challenges whose voices should be heard in designing children’s wellbeing measures. Several authors call for children’s perspectives to be used in the research (39–41). Biggeri et al. (39) and Fauth and Thompson (40) argue for a participatory approach where children are involved in both defining wellbeing and engaged in the research process.

In pursuing its aim to address these gaps in knowledge, this study asked the overarching research question:

How do schools use whole-school wellbeing promotion to enable pupils to flourish?

To answer this question as completely as possible, a series of sub-questions were developed to direct the research process:

1. What are the key components of whole-school wellbeing promotion?
2. How is wellbeing practice implemented and sustained?
3. What are the contextual factors which influence whole-school wellbeing promotion?
4. How do pupils experience whole-school wellbeing promotion?

## 2 Method

Ethical approval was obtained for this study from the University of Southampton.

### 2.1 Methodology

This article has been developed from the first author’s wider PhD project (42). Recognizing the multidisciplinary nature of this topic, the experience of the authors have brought a breadth of perspectives. The doctorate’s own healthcare background in occupational therapy was combined with the extensive expertise of the supervisory team in delivering and researching in the English education system, alongside the team’s own specialisms in health and wellbeing and learning outside the classroom.

The study adopted an exploratory approach to understand how primary schools designed, implemented, reviewed and sustained whole-school wellbeing promotion to enable pupils to flourish. A mixed methods (MMR) methodology was used (43, 44) to provide the most complete answer to the research question. Whilst traditionally MMR methodology has adopted more quantitative research methods, current discourse demonstrates a growing interest in a predominantly qualitative approach (45, 46). Thus, adopting mainly qualitative data collection and analysis methods in this project’s research design enabled the exploratory approach of the project (47). A qualitatively-driven mixed methods methodology may be defined as one where the theoretical ‘drive’ of the research is qualitative, with a greater weighting placed on this data type (46). As a result, the project was underpinned by the philosophical assumptions associated with qualitative research methods, namely, an interpretivist paradigm (47, 48). Alongside, the project ‘borrowed’ relevant quantitative methods as a ‘supplementary strategy’ to provide a more complete answer to the research questions than available through a wholly qualitative approach (46) (p. 3). A detailed presentation of the integration of results is available in the PhD thesis (42).

Interpretivist assumptions appeared appropriate for guiding the research design to capture and explore the perspectives of pupils, staff and school leaders and the influence of context (47, 49). To capture the subjective nature of people’s experiences spoken accounts, textual data, images and audio-visual artifacts on schools’ websites, as well as pupils’ drawings were used to collect rich, detailed data (48).

### 2.2 Theoretical framework

The study drew on three main theories: Seligman’s (4) PERMA model of wellbeing, WHO’s (50) health promoting schools (HPS) framework, and Bronfenbrenner’s (32) bioecological theory. The rationale being that whole-school wellbeing promotion was (1) a topic of interest across disciplines (18, 28, 35, 36), and (2) was inherently complex (51). It was concluded that no single theory could provide a complete understanding of the topic of whole-school wellbeing promotion, or adequately generate a comprehensive exploration of the research problem.

Seligman’s (4) PERMA model was used to provide a definition of wellbeing. WHO’s health promoting schools (HPS) framework helped to present the key structures and processes required in designing, implementing and reviewing whole-school wellbeing promotion (19, 52). Bronfenbrenner’s bioecological theory supported an understanding of a child’s embeddedness within their context, with the potential to understand the interrelationships between pupils, school, home and other environmental factors and their impact on wellbeing (48, 53). As each theory is widely used and has recognized underlying assumptions, their incorporation into the theoretical lens sought to provide credibility and confidence for the study (54). Furthermore, adopting three models mitigated the weaknesses of each and drew on their strengths.

### 2.3 The research design

**Figure 1:**
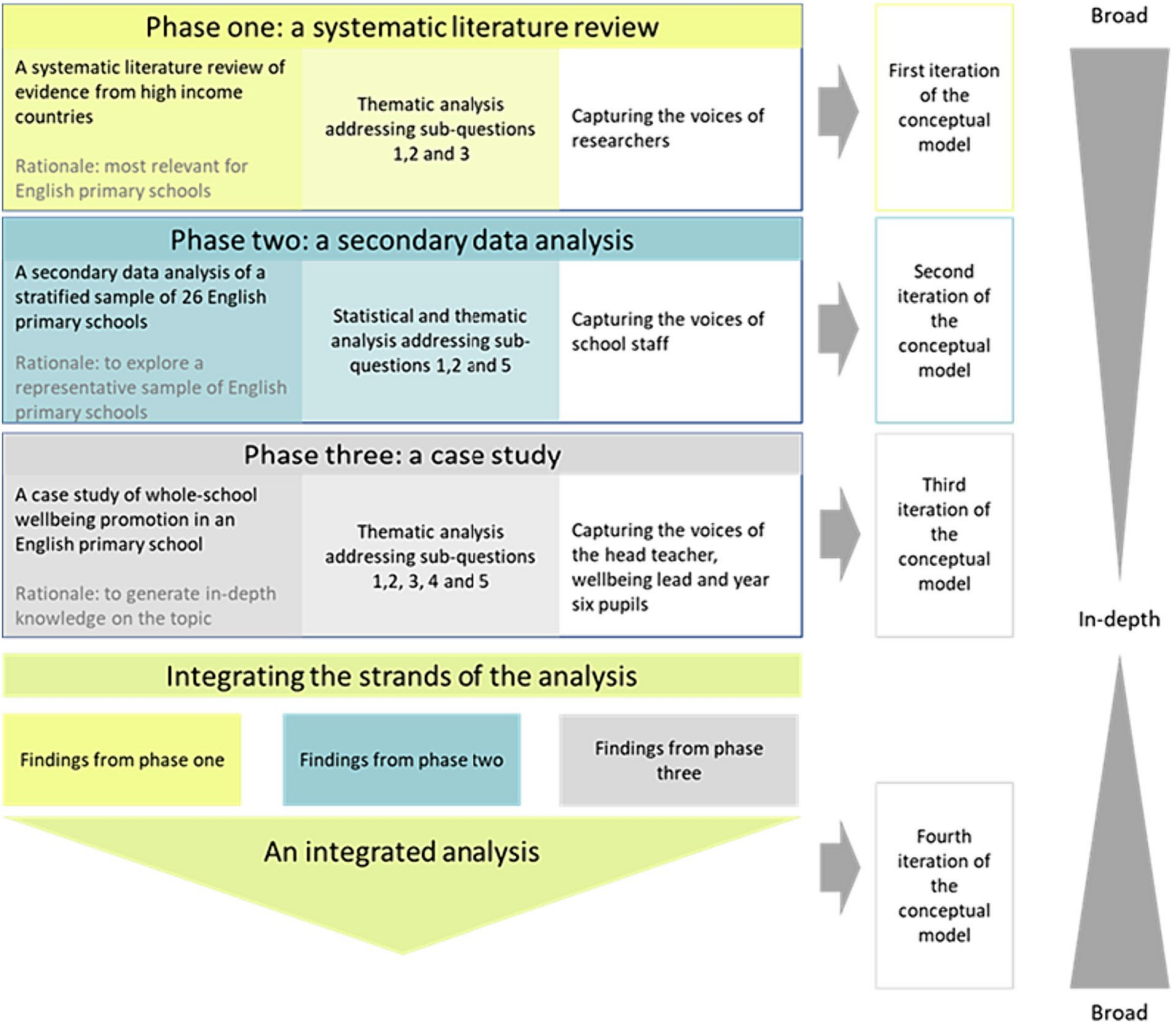
An outline of the research design.

A three-phase design was used to triangulate data from a range of research methods to provide a more complete exploration of the recognized complexities of whole-school wellbeing promotion (Figure 1) (43, 47). By analyzing the data after each phase, the findings iteratively informed the subsequent phases of the research design with the benefit of shaping the later phases to be most relevant to answering the research questions (54). The first author’s impact on the research process was reflexively considered, and supported by keeping ongoing notes (55).

#### 2.3.1 Phase one: a modified systematic literature review

A modified systematic literature review (SLR) was undertaken to provide a broad understanding of whole-school wellbeing promotion from an extensive body of international evidence. Its purpose was to establish whether common practices were adopted across high income countries and the resultant themes were used to inform and focus phases two and three of the study. Additionally, the themes were used to develop a taxonomy of deductive codes for the quantitative analysis of secondary data in phase two.

Literature was selected from high income countries as most relevant for the English education system. The purpose of this phase was to present a broad thematic analysis about how schools design, implement and review wellbeing practice with an aim of enabling pupils to flourish. Additionally, it assessed how factors in schools’ contexts promoted or hindered whole-school wellbeing promotion. Modifications to the systematic literature review protocol included the process being conducted by a single researcher and the review being completed over a 6 month period rather than the recommended longer timescale. To instill confidence in the study’s findings, it was important to be transparent about these adjustments.

##### 2.3.1.1 Procedure

###### 2.3.1.1.1 Search strategy

A systematic search for relevant research took place across seven databases: ERIC, Australian Education Index, CINAHL, MEDLINE, PsychINFO, SCOPUS and Web of Science. The terms “wellbeing or well being or well-being” OR “mental health promotion” OR “positive education” AND “whole-school or whole school” OR “universal” AND “intervention” OR “implement*” were searched in each database.

Teddlie and Tashakkori’s (44) 12-step framework for conducting a literature review was adopted as an overall procedural approach for the SLR, as this framework was produced by leading mixed methods theorists.

###### 2.3.1.1.2 Data collection

The PRISMA (Preferred Reporting Items for Systematic Reviews and Meta-Analyses) was used to guide this the systematic search (56). This provided an unbiased process through which to search for relevant literature to include in an SLR. Initial results were recorded on an Excel spreadsheet for each search term within each database (*n* = 901). Duplicates were removed (*n* = 382) and titles and abstracts were screened against the criteria. Where there was uncertainty, articles were included for full text reading to provide further clarity at the next stage. Forty-four papers remained at the end of this stage and were read in full at which point 24 were deemed ineligible. Twenty papers were included in the review. The Excel spreadsheets and final papers are included as supplementary information in the PhD thesis for transparency (42). The following inclusion and exclusion criteria were used:

Inclusion criteria

- Published in English.
- Between 2009 and 2019 (as most relevant for a rapidly changing research topic).
- In pre-school, primary and primary/secondary schools (ages 3–18).
- Universal interventions for all children and young people.
- Evidence from education, health sciences, psychology and sociology.
- Worldwide.
- Peer-reviewed empirical literature, book chapters, PhD theses or opinion pieces.
  Exclusion criteria
- Studies focusing on mental illness prevention.
- Interventions targeted to certain groups.
- General health promotion rather than promoting wellbeing (e.g., exercise, obesity, smoking).
- Focusing on secondary school only.

Hong et al.’s (57) mixed methods appraisal tool was used to assess the quality of publications. An Excel spreadsheet was developed to enable each paper to be assessed using the categories, questions and supplementary information in the MMAT version 2018 tool. As advised by the tool’s developers an overall score was not assessed, rather quality was determined by focusing on the detailed assessment provided by the tool.

###### 2.3.1.1.3 Data analysis

Braun and Clarke’s (58) widely tested reflective thematic analysis (RTA) framework was adopted to analyze the textual data from the selected published papers. It was chosen as a widely adopted method which ‘embraces qualitative research values’ making it appropriate for this qualitatively-driven project (59) (p. 333). RTA enabled patterns of meaning about concepts to be recognized both in relation to answering the research questions as well as highlighting important topics to the respondents and within the secondary datasets (58). This study’s analysis was predominantly data-driven using an inductive coding approach (58). However, some deductive codes were used to organize the findings in relation to the stages of planning, implementing and reviewing in whole-school wellbeing promotion. This reflects a common approach to using the RTA method, whereby scholars recognize some deductive coding is required to answer the research questions (60).

#### 2.3.2 Phase two: a secondary data analysis

**Figure 2:**
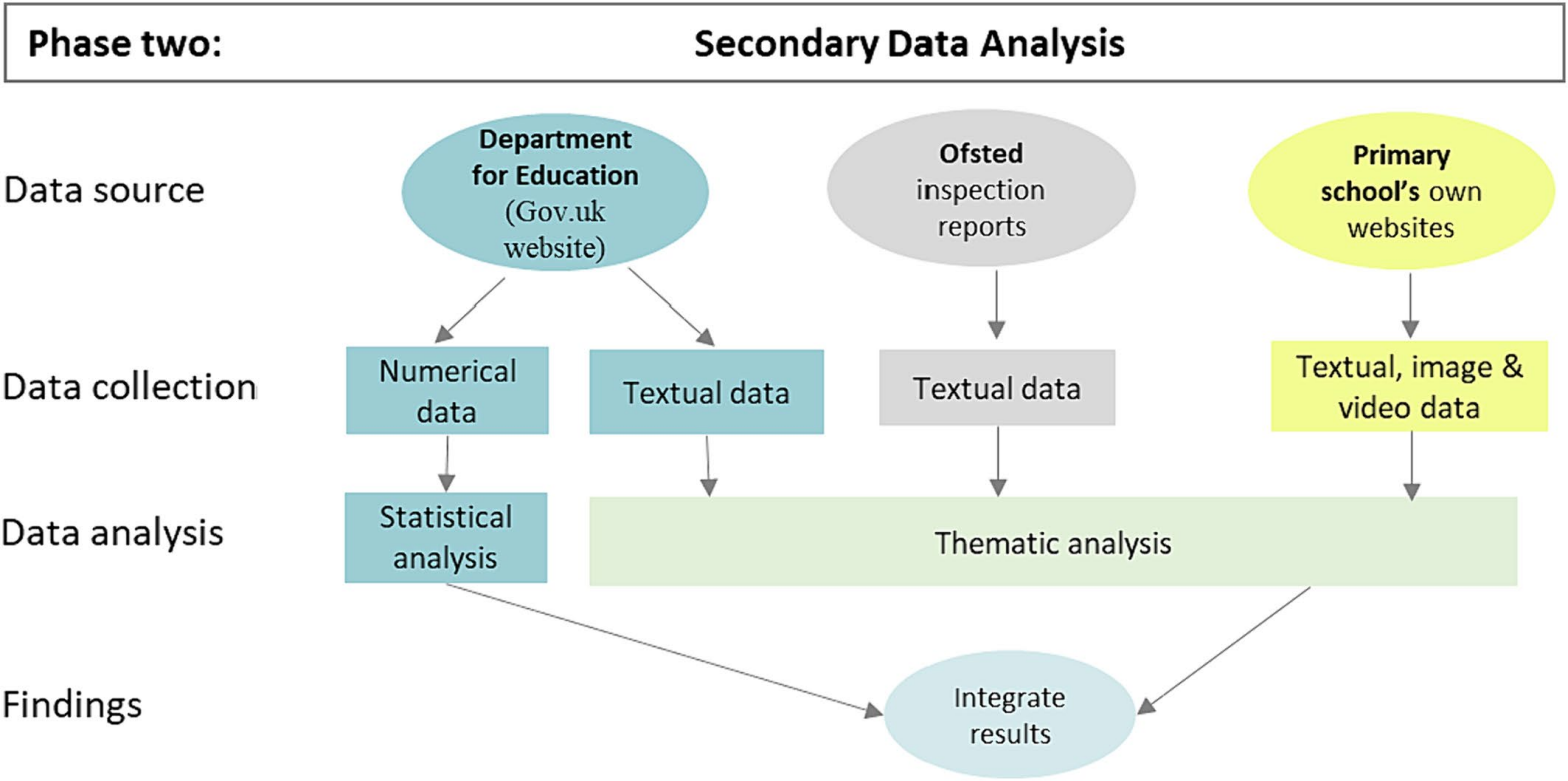
The secondary data analysis.

A secondary data analysis (SDA) focused on the English education system and explored how primary schools promoted pupils’ wellbeing (Figure 2). Despite a narrower focus than phase one, it still had the benefit of generating broad knowledge across a representative sample of schools. Its purpose was to collect and analyze qualitative and quantitative data using (1) a secondary dataset taken from the Department for Education publication on primary schools and (2) data from primary school websites within the public domain. As this existing data had been developed by different stakeholders for other purposes, it was deemed to be secondary data for this study. The findings complemented those of phase one by generating further evidence about the key components used by schools to design, implement and review whole-school wellbeing promotion.

##### 2.3.2.1 Procedure

###### 2.3.2.1.1 Sampling method

The population was defined as English state-funded, mainstream primary schools deemed to be undertaking whole-school wellbeing promotion to enable pupils to flourish. As there is no national list of schools promoting wellbeing, a suitable population needed to be identified. Several sources appeared appropriate. Firstly, a search identified schools recognized by third party organizations as demonstrating good practice. The nationally recognized wellbeing awards included the School Mental Health award (61), the Wellbeing Awards for Schools (62) and the Place 2 Be award (63), whereby schools were assessed on criteria for supporting wellbeing within their setting. Secondly, using Google, searches of school websites were made using the search terms (i) ‘wellbeing’ and ‘primary school,’ (ii) ‘mental health’ and ‘primary school’ and (iii) ‘positive education’ and ‘primary school’ and were limited to results within the first 10 pages to generate a manageable volume of results. This process yielded a study population of one hundred and thirty-five schools.

###### 2.3.2.1.2 Sample

A stratified sample of 26 schools was included in the SDA, the justification being that it allowed the analysis to capture a diverse array of school settings and characteristics, whilst enabling a manageable in-depth analysis (47). Of these schools, a purposive sample of 12 schools was selected for further qualitative analysis, chosen to represent a diversity of approaches and school characteristics within the overall sample.

A stratified sampling method was undertaken to gain a nationwide understanding of the topic by seeking to ensure representation of a wide-ranging selection of English primary schools (47). Stratification was based on a number of characteristics and informed by the sampling design used by Brown (64) in the DfE ‘Mental health and wellbeing provision in schools.’ Brown (64) (p. 8) justifies the strategy to ‘*ensure representativeness of schools with different characteristics and to limit the likelihood of biases*.’ This strategy was both relevant to the topic and already established in published research. Stratification was based on the following categories: geographical location, socioeconomic status, location (urban/rural), Ofsted (the national government school inspectorate) rating and school type. Brown’s (64) sampling strategy was modified to incorporate the latest government statistics on school demographics.

###### 2.3.2.1.3 Data collection

Data were captured during March and April 2020 during a period of school closure due to the COVID-19 pandemic. Sources included the Department for Education statistics, Ofsted inspection reports and schools’ websites. Qualitative data were imported into NVivo for thematic analysis, with additional hardcopies of relevant Ofsted reports and school webpages printed.

###### 2.3.2.1.4 Data analysis

The analysis comprised a qualitative thematic analysis and quantitative content analysis, with a larger weighting placed on the thematic analysis in line with a qualitatively-driven, mixed methods methodology. As with phase one, Braun and Clarke’s (58) RTA framework was used to guide the thematic analysis. Coding was inductively generated through immersion with the data. A smaller quantitative content analysis was undertaken using relevant textual and numerical data from all three datasets. A content analysis may be defined as the systematic search of material to uncover patterns of meaning through an analysis of concepts (65). Descriptive statistics were used to make sense of and present these findings (47). The content analysis was performed using an Excel workbook. Data were organized using the same deductive codes of planning, implementation and review as phase one. The statistical tools within Excel were used to sort and count the frequencies of codes.

Quality was improved through using guiding frameworks at each stage of the research process. Phase two also used robust datasets (66, 67). The DfE school statistics are produced in line with the Code of Practice for official statistics and Ofsted’s inspectorate adhere to quality assurance practices including clear and timely reporting using a guiding framework (68).

#### 2.3.3 Phase three: a case study

**Figure 3:**
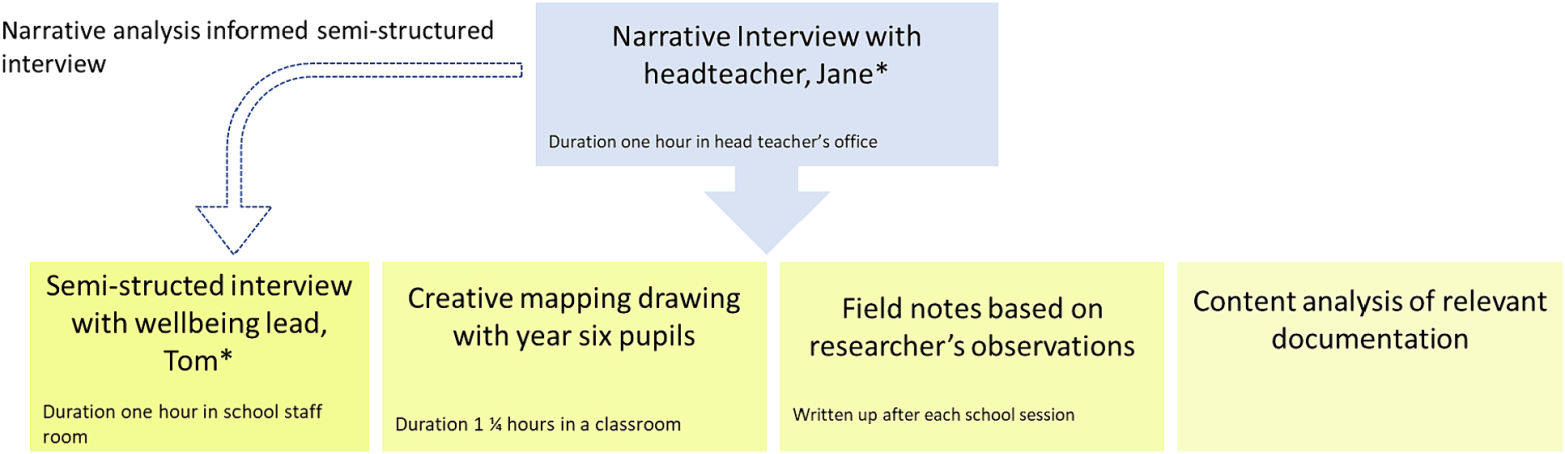
The case study design.

The case study was designed to provide a rich, detailed understanding of how whole-school wellbeing promotion was used in an English primary school to enable pupils to flourish (Figure 3) (69, 70). Due to the disruption of the COVID-19 pandemic resulting in periods of school closure, the original research design of a three-school comparison was amended to a single school. The limitations of this change and implications for further research are considered at the end of the paper.

##### 2.3.3.1 Procedure

###### 2.3.3.1.1 Sampling

An English primary school was purposively selected as a case of interest having undertaken whole-school wellbeing promotion for over two decades (54). The rationale being that this case provides an ‘*information-rich*’ sample through which to produce new knowledge (71) (p. 230).

###### 2.3.3.1.2 Data collection

**The narrative interview**

The narrative interview with the headteacher was completed in April 2021 in her office, lasting approximately 1 h and was guided by Jovchelovitch and Bauer’s (72) basic phases of the narrative interview framework. The interview was conducted by the first author and used a SQUIN (single question initiating narrative) structure, whereby a single prompt was used to enable the headteacher’s free flow narrative about designing, implementing, reviewing and sustaining whole-school wellbeing promotion to enable pupils to flourish (73). The prompt was as follows:

Thank you for agreeing to talk to me about your school’s journey, its biography if you like, of how pupils’ wellbeing has been and is promoted. My research aims to highlight how schools have undertaken their health and wellbeing journeys, and so I’d like to hear the story about what has happened at [name of school]. Please feel free to tell me anything your think is relevant. I do not want to be prescriptive as I understand each school’s story will be unique, but it seems as if there are some key features of their journey that schools have been through. These include the starting point, creating a vision, building enthusiasm, implementing changes, and keeping going. It would be really helpful if you could say something about how your school has moved through these stages, as well as what you consider to be the most important factors in promoting wellbeing and what has helped or hindered the process. Furthermore, the disruption of COVID-19 has had a major impact on both the wellbeing of pupils and the way in which schools operate, and I wonder how this has changed what you are doing. Overall, please tell me your school’s story about how pupils’ wellbeing is promoted.

Techniques of probing were used minimally to seek clarity. Two follow-up questions were used after the initial storytelling to uncover further details about the influence of context and the impact of the COVID-19 pandemic (72). Documentation referred to by the head teacher was printed out following the interview and used as part of the document analysis.

**The semi-structured interview**

The semi-structured interview was conducted between the first author and the school’s wellbeing lead (a class teacher) in June 2021 and lasted approximately an hour, in the empty staff room. An interview schedule was informed by the head teacher’s narrative interview, literature review, theoretical framework and findings from phase one. Some deviation from the schedule enabled areas of interest to be explored as they emerged (74).

The interview schedule was as follows:

What is the school’s overall vision for promoting wellbeing?

Which school policies relate to wellbeing?

What do you consider to be the most important components in promoting wellbeing?

Can you tell me the way in which your school uses the following factors to promote pupils’ wellbeing:

- The curriculum.
- The school’s ethos/culture.
- Non-teaching time such as playtimes.
- The part social environment plays.
- The part physical environment plays.
- Extra-curricular activities.
- Any other areas you consider important.

How do you measure the impact of what you are doing?

Within the school’s context, what has helped or hindered promoting pupils’ wellbeing?

How has COVID-19 impacted and changed what you do?

Is there anything else you would like to make me aware of?

**The mapping activity**

The mapping activity was undertaken in July 2021 with 17 year six pupils, enabling pupils’ subjective experiences of wellbeing and whole-school wellbeing promotion to be captured in an inclusive approach (75). The task lasted one and a quarter hours. The format of the activity was co-developed with the headteacher ahead of time and asked pupils to create maps of their wellbeing journey through their time at school.

###### 2.3.3.1.3 Data analysis

The interviews were transcribed verbatim using Microsoft Word, and stored anonymously (47). These transcripts, alongside the data extracted from the school documentation, were imported into NVivo software. Braun and Clarke’s (58) RTA method was used to inductively generate a series of codes and themes. Pupils’ maps incorporated textual and visual data, with textual analysis being carried out in the same method as above. Visual data were reviewed using Bland’s (76) process for analyzing children’s drawings. Analysis focused on ‘*the features given the most emphasis by the artist*’ (p. 238). Drawings and text from the maps were read concurrently for comparison of pupils’ sense-making (76, 77).

### 2.4 Integrating the strands of the analysis

The findings from all three phases were then integrated to provide a further analysis of the data in its entirety. This final analysis was designed to provide answers to the sub-questions and overarching research question, addressing the research problem and fulfilling the aim of the study.

In line with a mixed methods methodology, the strands of the analysis arising from the multiple research methods were integrated for meaningful knowledge construction (43, 44). Findings from each of the individual phases were written up and this text was imported into NVivo, with further inductive codes and themes generated. This integrated analysis forms the basis for the findings and discussion sections here, where the research question is considered in the light of this combined new knowledge.

## 3 Results

### 3.1 Characteristics of the data

This section presents the integrated findings from the three phases of the research design. The characteristics of included data are:

**Phase one: the modified systematic literature review**

The findings comprised 20 publications: six reviews (17, 18, 25, 28, 35, 36), 12 empirical studies (78–89), one protocol (90) and one opinion piece (91) (Table 1). Publications came from the Australia, Canada, Europe (Finland, Greece, Holland, Ireland and Norway) the UK and the US, and were published between 2009 and 2019. Eight papers were published between 2009 and 2012 and 12 papers since 2014. Seven studies were recently published between 2017 and 2019. The six reviews comprised three literature reviews, two systematic reviews and a meta-analysis. All reviews were published between 2011 and 2018, with two reviews coming from each of the UK, US and Australia. The majority of articles were empirical research (*n* = 12) of which six used a quantitative design, two used a qualitative design, two incorporated a mixed methods approach and two studies used case studies.

**Phase two: the secondary data analysis**

**Table 1:** The characteristics of papers included in the systematic literature review.

Total articles included in the SLR (*n* = 20)			
Category	Research design	Number of publications included (reference in brackets)	Countries
Review (*n* = 6)	Metanalysis	1 (17)	US (1)
	Systematic review	2 (18, 28)	Australia (1), UK (1)
	Literature review	3 (35, 36, 112)	Australia (1), UK (1), US (1)
Empirical studies (*n* = 12)	Quantitative	6 Quasi-experimental *n* = 4 (82, 83, 85, 87) Randomized control trial (RCT) *n* = 2 (79, 86)	Australia (2), Greece (1), Norway (1) Finland (1), Ireland (1)
	Qualitative	2 (81, 88)	Canada (1), UK (1)
	Mixed methods	2 (78, 89)	Netherlands (1), UK (1)
	Case studies	2 (80, 84)	Ireland (1), UK (1)
Protocol	Protocol for an RCT	1 (90)	Finland (1)
Opinion piece	Opinion piece	1 (91)	US (1)

Twenty-six English primary schools were included in the analysis. The representative sample was stratified on the following characteristics: region, location (urban/rural), type of school (maintained, academy, voluntary aided/controlled, free), school size (number of pupils), ethnic and cultural diversity (English as an additional language was used as a proxy), economic background of pupils (receipt of free school meals was used as a proxy) and Ofsted rating (school inspectorate).

**Phase three: the case study school**

The purposively selected school lies within the outskirts of a large city. It comprises approximately four hundred pupils between the ages of 4–11. Families have higher than average disadvantage with the number of pupils on free school meals in the last 6 years being roughly 40% higher than the national mean. Pupils where English is an additional language is about a third lower than the national average although the school’s families are culturally, ethnically and religiously diverse. The school’s location is within a built-up residential area close to a secondary school and sixth form college. Whole school wellbeing promotion was introduced to the school by the current headteacher over two decades ago. As a result, the case is an example of sustained, long-term wellbeing promotion which has been recognized to significantly benefit its pupils by the local authority, Ofsted, parents, governors and other external organizations. The case has been explored at a turning point where the long-term headteacher is leaving after over two decades of school leadership. Whilst it was unintentional to capture the cusp of this change, it has enabled the analysis to explore staff reflections on how wellbeing promotion may evolve as a result. This section refers to the headteacher as Jane and the wellbeing lead as Tom (not their actual names).

### 3.2 The development of six evidence-based principles in response to the overarching research question

The data collected at each phase were used to consider how schools planned, implemented, reviewed and sustained whole-school wellbeing promotion. In phases one and three, the way in which context shaped practice was also evaluated, and in phase three pupils’ own lived experiences of wellbeing practice were captured. Having organized and made sense of the data at each phase, the integrated analysis triangulated findings from all research methods to answer the research sub-questions. Original themes and sub-themes from each of the three sets of findings were subsumed into a final set of themes to address the questions (Table 2).

**Table 2:** Themes developed in the integrated analysis which answer the research sub-questions.

What are the key components of whole-school wellbeing promotion? (5 themes)	How is wellbeing practice implemented and sustained? (5 themes)	What are the contextual factors which promote or impede wellbeing initiatives? (3 themes)	How do children experience whole-school wellbeing promotion? (3 themes)
1. Vison and aims	1. Leadership and support	1. Schools’ social environments	1. Children’s experience of wellbeing
2. Approach	2. Embedding and formalizing practice	2. Other influencing factors within schools’ contexts	2. Challenges for whole-school wellbeing promotion
3. Design and content	3. Pedagogy for integrating wellbeing	3. The influence of the wider external context	3. The value of children’s experiences to inform
4. Additional opportunities	4. Reviewing outcomes and processes	–	–
5. Culture of wellbeing	5. Sustaining and evolving practice	–	–

**Figure 4:**
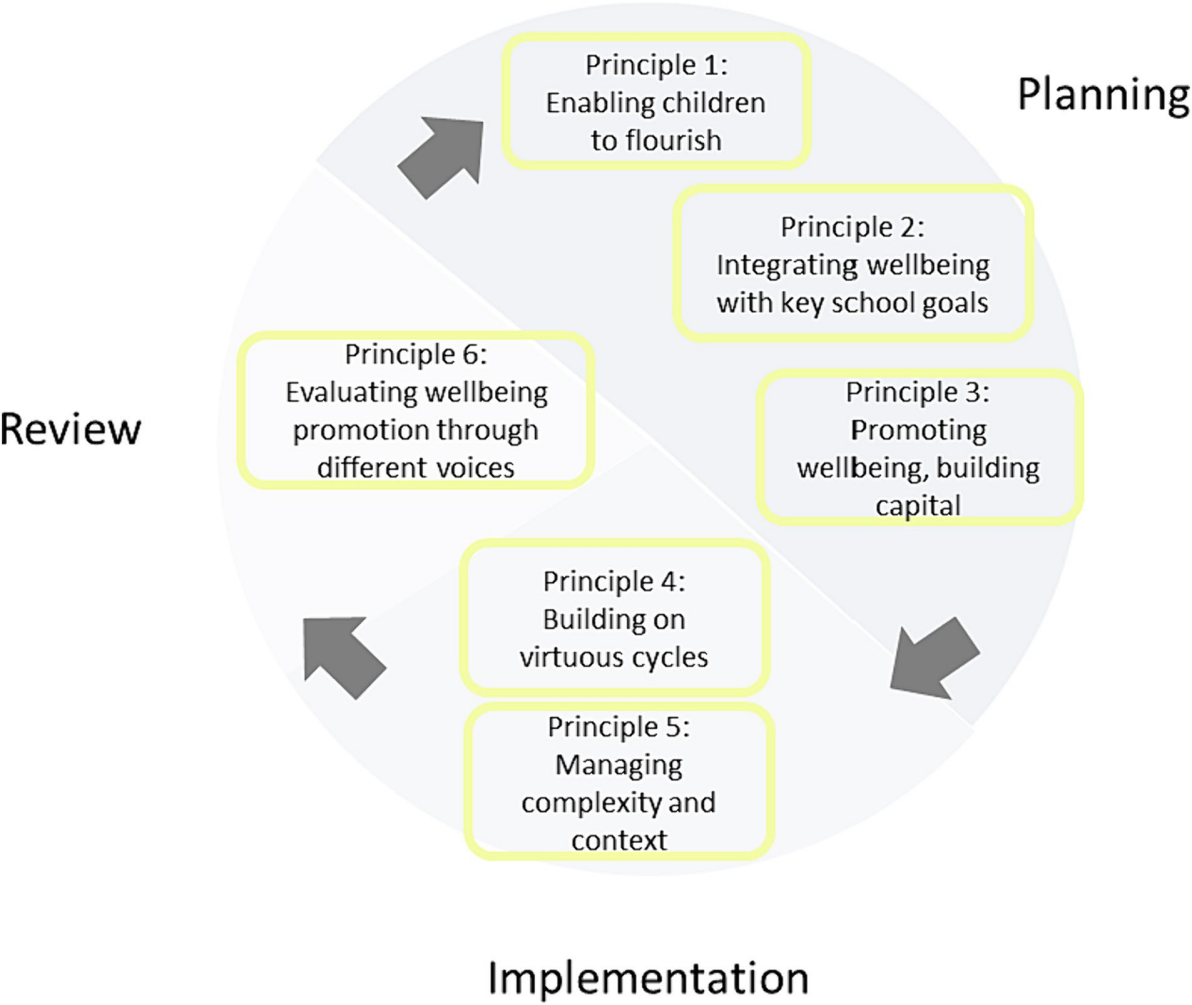
Six principles of whole-school wellbeing promotion.

To answer the overarching research question, **how do schools use whole-school wellbeing promotion to enable pupils to flourish?** a prolonged reflection on the integrated data was undertaken. This involved a process of stepping back and considering the analysis for several weeks, enabling consistent threads across the findings to become evident. These threads were translated into six broad principles (Figure 4): at the planning stage: (1) enabling children to flourish, (2) integrating wellbeing with key school goals and (3) promoting wellbeing, building capital; during implementation schools focused on (4) building on virtuous cycles and (5) managing complexity and context. In the reviewing process the analysis highlighted the importance of (6) evaluating wellbeing promotion through listening to different voices. These principles are predominantly derived from findings from phases 2 and 3 of the research as these relate to the English primary school setting. Findings from phase 1, the literature review, provide additional support.

### 3.3 Stage one: planning

#### 3.3.1 Principle one: enabling children to flourish

Schools understood the purpose of whole-school wellbeing promotion was to develop and sustain high levels of wellbeing amongst their pupils, with most schools in phase two, the SDA, aiming for pupils to ‘*flourish*’ by developing multiple salutogenic sub-goals. Frequently cited aims included building positive relationships (*n* = 26), positive mental health and wellbeing (*n* = 26), learning and achieving (*n* = 26), strong home-school partnerships (*n* = 22), developing confidence and self-worth (*n* = 22), being happy (*n* = 20), celebrating uniqueness (*n* = 20) and building resilience (*n* = 15). On average schools stated between 12 and 13 wellbeing aims. In contrast, the case study school understood that promoting pupils to flourish mediated ‘*offsetting disadvantage*,’ experienced widely within their catchment area. In addition, 15 schools in phase two aimed to promote staff wellbeing and 12 to support family wellbeing. The importance placed on developing a whole-school culture of wellbeing was echoed by Tom who emphasized that staff wellbeing led not only to *‘personal fulfill*ment’ but also ‘*benefit[ted] our pupils*.’ As a result of the school culture year six pupils recognized that the school’s wellbeing practices supported them both to enjoy their time at school as well as overcome a variety of social, learning and situational challenges. Children used a range of vocabulary to capture day to day feelings about school including being ‘*happy*’ and ‘*safe*,’ feeling ‘*proud*,’ ‘*loving*,’ *‘excited*,’ ‘*fun*,’ ‘*enjoying*,’ ‘*joking*’ and ‘*brave*.’ In addition, several children reflected on the benefits of accomplishment through feeling more ‘*confident*’ and ‘*proud*,’ with two children highlighting particular enjoyment in mastering personally meaningful subjects of reading and math over the longer-term. This suggests that pupils understood that flourishing required a combination of hedonic (pleasurable) and eudemonic (meaningful, satisfying, goal oriented) experiences.

**Figure 5:**
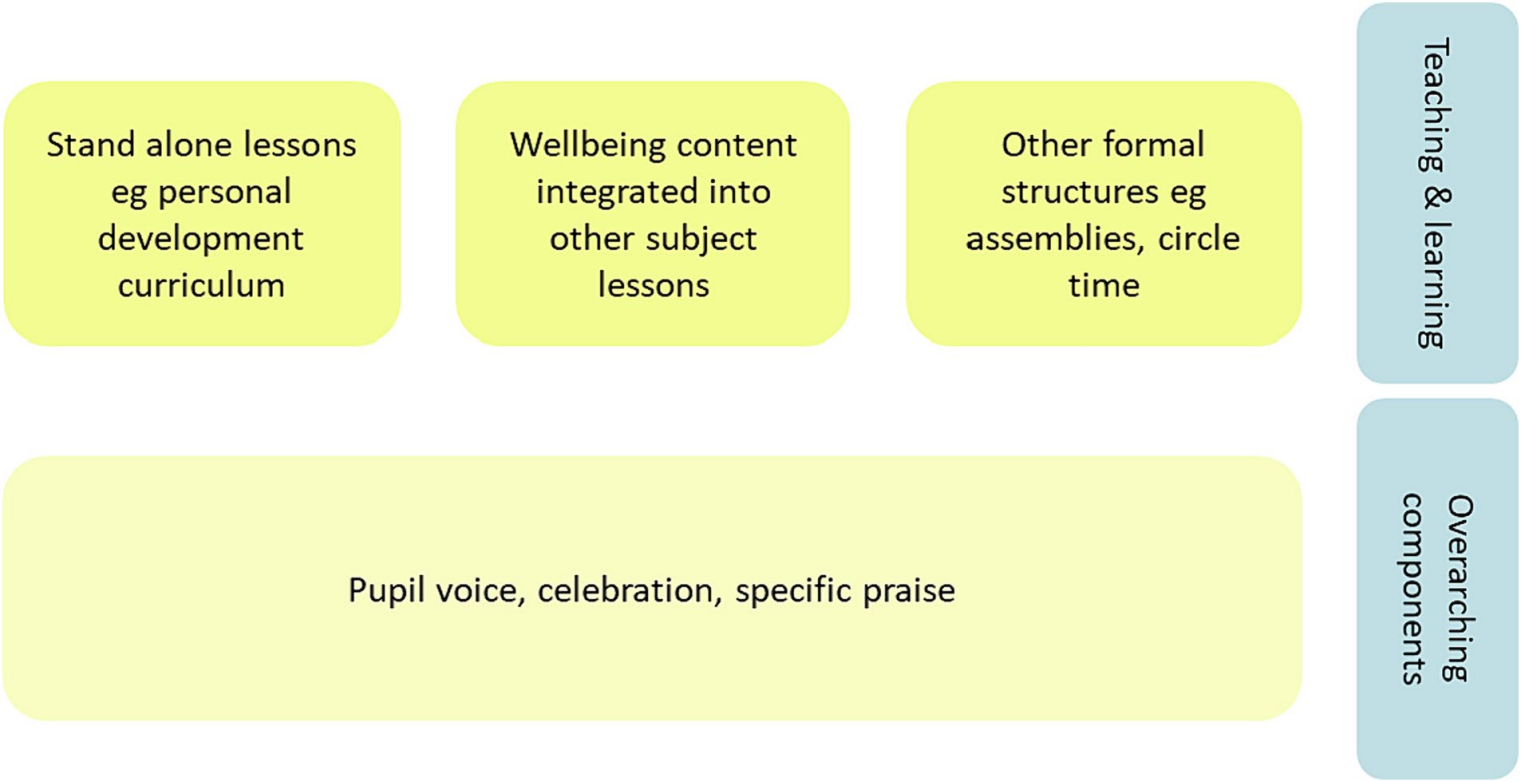
Common components in whole-school wellbeing promotion.

This multi-faceted approach was evident in the diversity of strategies and evidence-based underpinnings adopted by schools (Figure 5). In both phases two and three use of a whole-school approach was evident through complex, multi-strategy designs, adopting formal lesson content, other formal structures and informal staff-pupil social interactions. Whilst schools used unique approaches to combine strategies to best address their local setting, there was evidence that rather than ‘reinventing the wheel’ some schools adopted existing wellbeing practices through adopting (1) evidence-based approaches (*n* = 14), (2) provision by external organizations (*n* = 17), and (3) the criteria for obtaining an award (*n* = 4). Schools also demonstrated that they adapted existing resources for their own setting. In the case study school, Jane highlighted how they adopted a leading UK mental health charity’s questionnaire for their annual wellbeing survey for pupils and parents, which was adapted in the light of families wellbeing concerns during the COVID-19 pandemic.

All schools in phases two and three used standalone lessons, known as personal development lessons (PDL) or personal, social and health education (PSHE) lessons, to promote skills and knowledge related to social and emotional learning. Content was also integrated into other curricula with Tom evidencing how discussions related to wellbeing were incorporated into English and history lessons:

*‘… Shackeleton exploring the Antarctic. I am looking at the advert where he said we want men for this. And that turned into quite a long discussion about gender and … would that be acceptable today? (Tom).*

There was evidence that enabling pupils to flourish occurred at different levels: personal interactions, classroom-level circle times which enabled pupils to raise issues of concern and whole-school level assemblies where celebrations took place. All schools recognized these multi-level structures enabled formal and informal interactions between pupils’ peers, teaching staff and school management which enabled pupil voices to be heard. In the case study school, the rich description enabled additional strategies to emerge. Jane used the ‘*issue of the month*’ to enable all pupils to discuss issues of personal importance during assemblies:

‘… *through this, you know, a child will bring the views of the class to, then, a meeting relating to a particular issue, and that issue changes through time … and often those issues do relate to wellbeing …*’

In addition, echoing the SDA schools, she advocated for celebration as a mechanism of giving pupils agency of their own wellbeing: *lots of celebration - I think that's really important … ways hopefully that are, that are empowering and helpful and we do quite a lot around, you know what does encouragement look like, to enable children to build their self-esteem*.’

Pupils recognized the benefits of celebration, reporting that the school’s reward systems were both enjoyable and satisfying:

‘*I was proud of myself because I was mostly on the sun [reward system]*.’ (Child 10, reflecting on year 1).

Another mechanism used to mediate agency was the use of specific praise:

*‘I really like the way you spoke to me just then’ so the children actually can latch on to what it is that was good, and then to build on more of that … to make long term improvements in their self-esteem rather than quick fixes …*’ (Jane).

This is a further example of Jane’s belief in the importance in transferring validation from the teacher to the child. It implies her vision that flourishing is improved where children are encouraged to be autonomous agents who engage with wellbeing promotion, rather than passive receivers of the initiative.

#### 3.3.2 Principle two: integrating wellbeing with key school goals

All schools recognized the interrelationship between wellbeing and learning, highlighting how high levels of wellbeing resulted in improved readiness to learn and attainment. The SDA demonstrated schools typically focused on several values to develop a supportive culture for learning and wellbeing. Respect (54% of schools), kindness (38%), responsibility (27%), tolerance (27%) and aspiration (23%) were cited most frequently. In the SLR schools sustained a positive culture through embedding these values in the school ‘rules.’ In Elfrink et al.’s (83) study, schools adopted ‘*life rules*’ such as ‘*People get happy when I give them a compliment*.’ Similarly, the case study school values were embedded in the ‘*Golden rules*.’ Five schools in the SDA used the evidence-based Values-based Education framework and faith schools implemented religious values. Schools understood culture as a method to facilitate pupils’ agency and readiness to learn and achieve.

As an alternative strategy, some schools developed an overarching strategic vision which placed equal importance on both learning and wellbeing as key school goals. Illustrations included one school using a ‘*wellbeing system*’ alongside academic ‘*excellence*’ and another focusing on social, emotional and academic development equally. This approach implies that these schools recognized their role as focusing on the holistic development of pupils. Therefore, key goals were viewed as wider than academic learning and performance, additionally encompassing social, emotional, psychological and physical aspects of pupils’ development.

#### 3.3.3 Principle three: promoting wellbeing, building capital

All schools understood the benefits of offering pupils extracurricular activities and other opportunities for their wellbeing, with a smaller number recognizing a relationship between promoting wellbeing and building cultural and social capital. In the SDA, schools promoted wellbeing through residential visit and outdoor learning. Strategies focused, predominantly, on eudemonic aspects of wellbeing including meaningful challenges, risk-taking and rewards, and engaging pupils through goal setting. In the case study school, year six pupils emphasized how these opportunities enabled them to develop skills, confidence, self-esteem and independence.

In England, schools take pupils away, typically for a week, to a different setting where they are involved in a range of organized activities. This includes staying away from home with staff and peers within their year group. A pupil reflecting on the year four residential trip recalled:

‘*we went to [name of residential trip] I was proud because I was scared to go but I went*’ (child 10, reflecting on year 4).

The findings also demonstrated how schools expanded an activity to provide pupils with extra opportunities to enhance wellbeing. One school in the SDA used space in a community allotment for gardening. This enabled children to foster relationships with members of their local community, thus widening their social network. In another school the produce from their gardening activities was sold on the school playground, evoking a sense of satisfaction in pupils selling their own food alongside promoting belonging in the local community. In a third school pupils were given a sense of satisfaction from using gardening to benefit of the local area by developing a community garden in collaboration with a gardening charity. These findings also implied that schools recognized that using existing activities such as gardening undertaken for the science curriculum, had the potential to improve wellbeing simultaneously, and is another example of not needing to ‘reinvent the wheel’ to promote wellbeing.

Two schools in the SDA and the case study school served areas of greater than average disadvantage and had a focus on how providing pupils with a range of life experiences could support them to flourish better in the longer-term. Jane emphasized how the vision was to ‘*offset disadvantage*’ by normalizing diverse experiences to broaden pupils’ and families’ horizons, to improve longer-term wellbeing: ‘*widen [ing] children’s experiences – trips, visitors, opportunities to learn outside the classroom*’ (PDL curriculum). Both Jane and Tom recognized that these opportunities both improved wellbeing and increased cultural and social capital:

*‘This is a school that actually takes the arts seriously … it’s encouraging that creativity … giving the children cultural capital … [Art and music] really are a part of developing wellbeing’ (Tom).*

The two other schools identified their role as building foundations for lifelong wellbeing. One school focused on making children ‘*life ready*’ for adulthood and the other emphasized how life skills can be taught at any age. Both schools offered extensive programs of developing life skills for the longer-term, providing a range of opportunities outside regular teaching and learning. School N had developed a mind, body and soul approach with a focus on ‘*academic achievement, physical development and positive wellbeing*.’ It achieved this through engaging pupils in 70 character-building experiences and developing 80 key life skills to ensure pupils were ‘*life-ready*.’ School F focused on developing the school environment to offer a range of bespoke opportunities. Leasing extensive grounds enabled pupils to undertake animal husbandry on the school farm and engage in outdoor pursuits, an artist in residence supported pupils’ creativity and children had access to recording resources for music and school radio. Both schools had been recognized through national awards, including ‘the happiest primary school in Britain,’ Place2Be award and TES awards.

### 3.4 Stage two: implementation

#### 3.4.1 Principle four: building on virtuous cycles

A virtuous cycle is defined as a series of incremental changes which synergistically improve outcomes and the Department for Education (92) proposes that addressing the ‘physical and psychosocial well-beings’ enables pupils to ‘achieve better academically, which in turn leads to greater success’(p. 3). Schools understood the importance of implementation in sustaining whole-school wellbeing promotion. In the SLR five reviews and 6 empirical studies considered how implementation influenced the overall effectiveness of whole-school wellbeing promotion, reporting the importance of high-quality implementation (17, 25, 28, 35, 36, 78, 80–83, 88). Durlak et al. (17) and Weare and Nind (18) proposed that the higher the quality of implementation, the greater the positive effects. This was echoed by Banerjee, Weare and Farr (78) who concluded that where implementation of the SEAL program was rated highly, children adopted more effective learning strategies and experienced greater levels of motivation. Furthermore, Dix et al. (81), in assessing the outcomes of the Kidsmatter program across 96 Australian primary schools identified that where implementation was rated highly Year 6 pupils’ academic achievement was 6.2 months ahead of those where program implementation was poorly rated. Moreover, Omstead et al. (88) concluded that where implementation was of high quality this cultivated enthusiasm amongst staff and pupils, a key component of long-term sustainability. In the case study school Jane recognized that improving wellbeing was a process of continual change, requiring regular review and refinement to enable pupils to flourish. Jane perceived value in ‘*process*’ versus the ‘*product*,’ illustrating that achieving a wellbeing award (the product) was less valuable than the living, sustained process of promoting wellbeing.

Leadership style was perceived as an important component of driving sustained practice. In the SLR Holsen, Iversen and Smith (85) recognized that strong leadership enabled more consistent implementation of a SEL intervention across classes. Omstead et al. (88) highlighted that schools with committed, engaged leadership were more successful in developing cultures where pupils felt safe, connected and valued. Similarly, Jane perceived her ‘*passionate*’ leadership style promoted a supportive culture to meet the wellbeing needs of pupils, staff and parents.

In the case study’s rich description Jane alluded to traits she recognized in good leadership, including ‘walking the talk’ which she exemplified by following the school’s golden rules of ‘*honesty*’ and not ‘*cover [ing] up the truth.’* She also valued recognizing your limitations ‘…*knowing that you cannot do it on your own… it’s knowing when to go to others as well’* and *[Being a] reflective practitioner is massively important to that [promoting wellbeing].*’ Distributed leadership was also seen as central to staff and pupil wellbeing. Tom identified that *‘We are trusted in our professional judgement about what helps the children the best.*’ Tom’s comments also imply a sense of continual change as children’s needs change and new cohorts come into their classrooms.

Schools also understood communicating a shared vision is essential for sustaining practice. Banerjee, Weare and Farr (78) and Dix et al. (81) recognized the importance of early communication with all stakeholders, including parents as a mechanism for ‘buy-in,’ that engages the whole school community. The benefit of communication enabled schools to establish wellbeing practice as a key component of school ‘*business*’ (79, 81). The case study school focused on the importance of ongoing communication. Jane perceived a common wellbeing language as essential for consistent communication within the school community, ‘*I can set that tone… in the wording that I use, the language that I use… And that’s really important in… the cohesion around wellbeing as well.*’ Tom understood sustained practice was driven through ‘*keeping it on the agenda.*’ He argued conversations between staff and children alongside communications through regularly changing displays were vital to their whole-school approach.

Schools recognized practice evolved over time. Jane identified that change was not consistent but resulted from periods of rapid change, such as during her arrival at the school, followed by slower progress whilst embedding practice across school life. Evidence of this was found in Omstead et al.’s (88) study of whole-school change where two schools with rapid staff turnover realized far fewer benefits for their pupils.

#### 3.4.2 Principle five: managing complexity and context

Findings across all three phases demonstrated the complex nature of wellbeing promotion, evidencing the volume and diversity of visions, aims and strategies schools adopted as well as the contextual factors which promoted and hindered practice. In addition, Jane alluded to the challenges of addressing differing pupil needs:

‘… *recognis [ing] that rights conflict … rights of the, the one child … and how you [balance that] with the rights of all children… being honest about that rights conflict…*’

Year six pupils highlighted how subjective wellbeing was influenced uniquely, with 2 year six children demonstrating a teacher’s behavior promoted happiness in one child but had a negative effect on another pupil. As a result, schools provided pupils with a range of pedagogic learning experiences to address the uniqueness of children’s responses to wellbeing promotion. These included playing games, simulations, modeling positive behaviors, using discussions, open-ended questioning, role play and storytelling, enabling pupils to practise their learning within a supportive culture, incorporating questioning, self-reflection, problem solving, critical thinking and solution-focused decision-making. Schools recognized this to enable children to be actively involved in their learning.

Findings also identified that characteristics of the pupils themselves were influential on how wellbeing was promoted. Children’s anxiety was a potential barrier to their subjective sense of wellbeing, with many schools seeking to adapt their environments to mitigate perceived challenges. Almost half of schools had introduced eco activities such as enabling pupils to recycle waste and achieving nationally recognized eco awards. Schools understood this facilitated children’s sense of wellbeing through having agency in looking after their environment. Most schools offered alternative activities alongside traditional unstructured play and provision of large equipment supporting physical play. One year six pupil described feeling happy because ‘*the headteacher lets you do colouring at lunch if you do not want to play outside*.’ A small number of schools had adopted calm clubs and spaces for children seeking quieter play or solitary time at lunchtimes. Schools differed as to whether children were selected to attend these alternative activities or whether sessions were open to all children.

The case study school exemplified how the interrelationship between contextual factors affected children’s ability to flourish. Jane recognized that addressing parents’ anxieties respectfully enabled pupils to engage in beneficial extracurricular activities:

*‘if a parent is, is anxious about their child going out … we don't just say ‘Oh, ok well that child will sit in the corridor for the day’ - it's about ‘ok so what can we do to reduce your anxieties around this?’ How can we support and help…respecting parents views is really important.*’

This is echoed in the SLR by Clarke, O’Sullivan and Barry’s (80) findings about differences between two schools both identified as ‘disadvantaged,’ one in an established, supportive community, and another with a transient population from traveling families with few parent or community links. Parents of the first school perceived the social and emotional learning program as more beneficial and provided higher levels of skills practice at home.

There was also evidence that school-level issues were driven by macro-level factors. Several schools Identified lack of teacher time as a limiting factor to promoting wellbeing (80, 83). Similarly, Tom highlighted how political decisions resulted in an overly long waiting list for the emotional literacy support assistant in the case study school. This discussion emphasizes not just which factors influence local school practices but also the complexity of interactions between factors. Despite a growing governmental focus on wellbeing in English schools, there is restricted funding for schools as part of wider economic policies. Thus, the government acts as both facilitator and barrier to broadening schools’ involvement in promoting wellbeing.

### 3.5 Stage three: review

#### 3.5.1 Principle six: evaluating wellbeing promotion through different voices

The outcomes of whole-school wellbeing promotion was measured both formally and informally. In the SDA, formal methods included review by external organizations and internal pupil, parent and staff surveys. External reviewers included Ofsted inspectors (the schools inspectorate) and assessed by awarding institutions including the Wellbeing for Schools award and Carnegie School Mental Health award. Other bodies included Values-Based Education, Peaceful Schools, the Inclusion Quality Mark as well as one-off awards such as the DfE Character Award, the ‘Happiest Primary School’ (in the National Happiness Awards) and TES (Times Educational Supplement) school awards.

The most used method was informal parental feedback on how they perceived pupils’ wellbeing (*n* = 15). Fewer schools demonstrated how pupils were consulted. Nine schools encouraged pupil feedback through facilitating an open-door policy or drop-in sessions. Other evidence schools included interactions with wellbeing champions and ambassadors (*n* = 6), pupil questionnaires (*n* = 5), feedback from the provision of reflection times (*n* = 6), the use of worry boxes/monsters (*n* = 6) and traffic light or mood cards to capture a snapshot of children’s feelings (*n* = 3). Teachers made use of life skills learning as opportunities to observe pupils’ skills development (*n* = 2).

One school highlighted the importance of facilitating pupils to self-evaluate wellbeing, with year 2 and 4 children reflecting on activities to feel better and breathing techniques for emotional regulation. The case study school demonstrated how information collected was used in the long and short term. Aggregated survey responses were used for longer-term, strategic decision-making:

‘*we do a pupil impact survey each reflecting on year … [questions like] being optimistic for the future… anxieties and hope, things like that … where are you at really?*’ (Tom).

Whereas, at a pupil level, reward systems were used to identify individual, short-term needs:

*‘we keep track of who’s maybe not earning their golden time and if there’s a pattern. If someone is clearly having a difficult time that would be something that we could look into’ (Tom).*

The analysis sought to explore how whole-school wellbeing promotion was experienced by sub-groups of pupils. In terms of gender, only two schools in the SDA ran sessions for boys about the role of the man in society, and no activities were offered solely for girls. No schools provided evidence that they measured wellbeing outcomes by gender, although a lack of findings may be attributable to this study’s research design, in part, using a secondary data analysis on existing datasets (67). The findings have, however, generated new knowledge about how English primary schools recognized the impact of disadvantage on their pupils’ wellbeing, with the potential for pupils from lower socio-economic status (SES) families less likely to flourish due to limited positive opportunities and experiences and more negative contextual influences. There was also evidence that schools used a longer-term vision to meet the additional needs of pupils from low SES backgrounds in supporting those pupils to flourish. These schools identified the importance of building foundations to improve wellbeing in adulthood and to widen pupils’ and families’ lifelong horizons (as outlined in principle three).

A further sub-group was looked-after children (who lived in care). Jane emphasized how this group had the potential to feel isolated. The school recognized that facilitating these pupils to identify their ‘tribes’ promoted a beneficial sense of connection.

*we don't make a group of looked after children but actually enabling children to know that they are not the only child that doesn't live with their you know their birth parents in school is a really helpful thing* (Jane).

The study was less able to capture the experiences of pupils with special educational needs. In the case study school with a relatively large number of pupils in this group, a single voice was captured through the mapping activity. The environment was modified to enable the child to engage in the task alongside their peers. They were accompanied by their learning support assistant and sat in a specially modified corner of the classroom. Whilst, their perceptions of wellbeing practice were aggregated with other pupil voices and thus unable to be highlighted in this analysis, the successful completion of the mapping activity demonstrated that the research method had the benefit of being inclusive for all pupils in the school.

## 4 Discussion

This section uses the six principles once more to situate this study in a wider context, to highlight this study’s original contribution and emphasize areas for further research.

### 4.1 Enabling children to flourish

The study recognizes ‘flourishing’ as experiencing high levels of wellbeing across five dimensions: positive emotions, engagement, relationships, meaning and achievement (4). Seligman’s model incorporates both hedonia, immediate, short-lived experiences associated with positive emotions, and eudaimonia, which is related to satisfaction and develops over the longer-term (4, 5). Thus, it was appropriate that the findings demonstrated that schools focused on providing pupils with a range of opportunities to experience both aspects of wellbeing.

Flourishing is associated with children enjoying life and coping better with adversities (5). Particularly schools serving disadvantaged areas, demonstrated a focus on both short-term and longer-term wellbeing benefits which resonates with Ben-Arieh and Frones’ (31) (p. 463) concept of children ‘*being*,’ and ‘*becoming*.’ Findings reflect their language of children experiencing ‘*wellbeing*’ and ‘*well becoming*.’ The analysis revealed a diverse plethora of visions, aims and strategies that schools, as ‘*social structures*’ use to shape ‘*the unfolding of the life course*’ of their pupils (p. 463).

**Figure 6:**
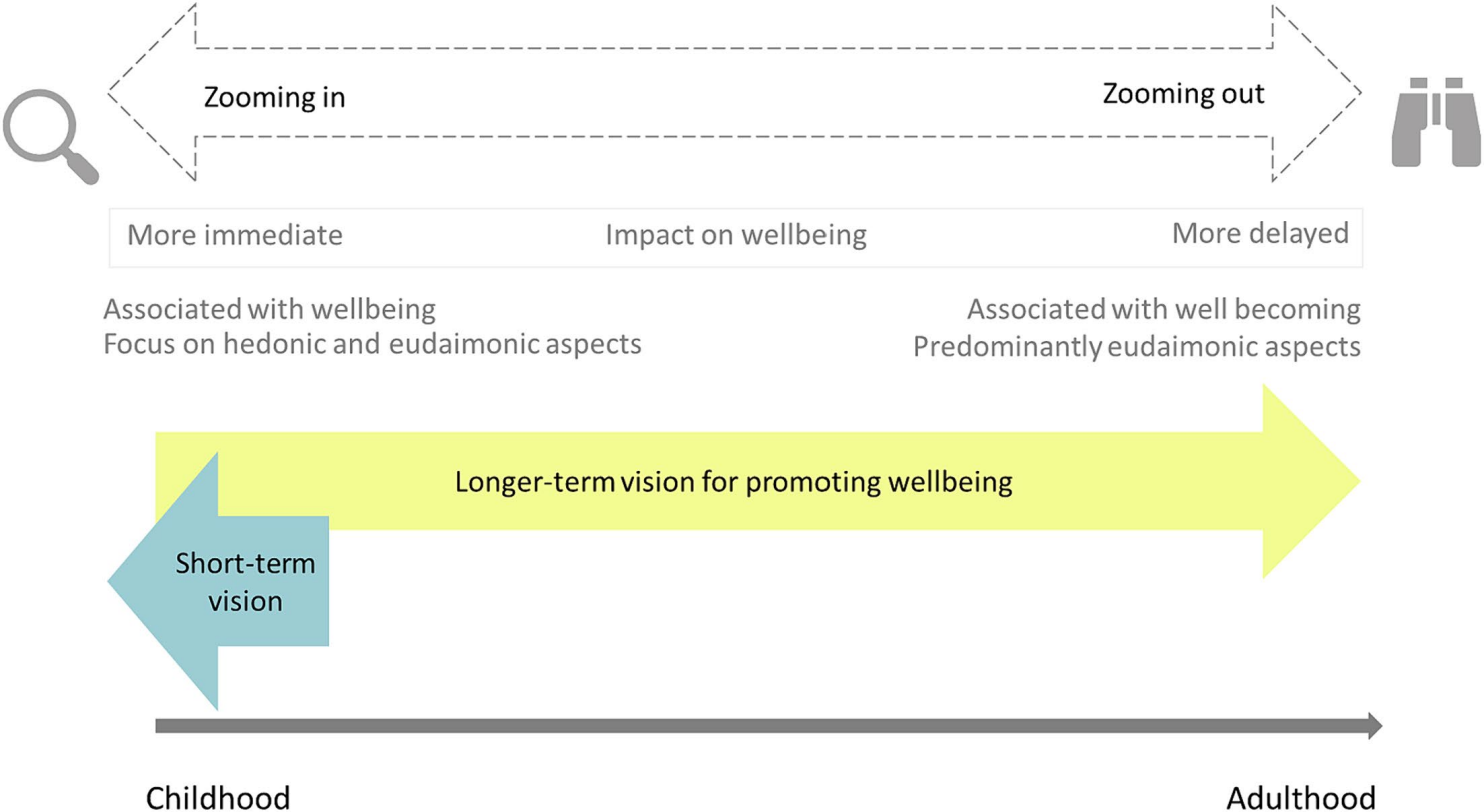
The zoom aspect of whole-school wellbeing promotion.

It appeared that schools, therefore, use a ‘*zoom aspect*’ in their approach to promoting pupils’ wellbeing (Figure 6). Schools ‘*zoomed out*’ to incorporate opportunities for all pupils to gain skills and experiences associated with longer-term benefits such as resilience, problem-solving and building self-esteem and self-worth. Children emphasized how these strategies promoted a sense of lasting satisfaction, pride in themselves and self-efficacy as agents of their own wellbeing. The zoom aspect resonates with Ben-Arieh and Frones (31) concept of well becoming. In contrast, ‘*zooming in*’ describes the process whereby schools met pupils’ short-term wellbeing needs, through promoting the development and practice of skills and knowledge for immediate social, emotional and psychological wellbeing within a supportive school culture, reflecting the concept of ‘wellbeing.’

### 4.2 Integrating wellbeing with key school goals

Whilst education policy and the school inspectorate are promoting personal development and pupil wellbeing as a sector goal, its implementation takes place at individual school level. Integrating wellbeing as a key goal may be perceived as challenging for schools unless its benefits are fully understood, particularly without the provision of additional resources. Many schools recognized that high levels of wellbeing positively benefitted pupils’ learning. This reflects Public Health England’s [(93, p. 7)] judgment that wellbeing promotion which develops social and emotional skills alongside positive changes to the ‘*culture, ethos and environment of a school*’ facilitates the largest improvements to pupils’ educational performance. Yet it also raises a broader concern over workload, evident in the Teacher Workload Survey (94) which reports that 52% of primary teachers perceive workload as ‘*a fairly serious problem*,’ implying constraints on time available for promoting pupils’ wellbeing (p. 5). This has been further exacerbated by the disruptions of the COVID-19 epidemic, with a resultant decline in pupil wellbeing (95). Thus, it can be argued that unless wellbeing promotion can be synthesized with schools’ key goals it has the potential to be seen as an additional burden for school staff. Historically the purpose of education has focused on learning and attainment, although DfE (2019) now reports that a further goal for schools is to build character, resilience and wellbeing. The findings suggested that whole-school wellbeing promotion can reinforce these key goals in two ways: (1) by driving improvements in academic performance, the traditional role of education and/or (2) by fulfilling the wider remit of providing personal development, identified as a key goal of education in the latest inspection framework for English schools (68). These findings concur with those of other researchers who found promoting the skills associated with wellbeing mediates academic improvements (96–98). Thus, a benefit of this study appears to have been the useful real-world knowledge about how schools integrate wellbeing promotion with learning in the English education system.

### 4.3 Promoting wellbeing, building capital

The case study school emphasized the importance of building pupils’ capital as a way to offset disadvantage. Bourdieu’s (99) concept of cultural capital is associated with a person’s assets in terms of their knowledge, preferences, interests, possessions and education. He argued that cultural capital leads to inequalities, particularly for those with lower socio-economic status whose assets restrict opportunities within society. In response, Ofsted now requires schools to facilitate cultural capital as part of pupils’ personal development (100). This study identified that schools recognized a positive link between promoting wellbeing and building cultural capital, with both being acknowledged to offset inherent social and health-based inequalities (99, 100). Additionally, the analysis found that social capital was facilitated through schools’ wellbeing practices such as expanding the activity of gardening to promote opportunities for social engagement in the local community. Bourdieu (99) defined social capital as the development of social cohesion, belonging and involvement in social networks.

This gives further weight to the argument that wellbeing and pupils’ capital are closely related, and that schools can facilitate both simultaneously through existing school activities. This echoes Portela et al.’s (101) (p. 1) study of the links between social capital and subjective wellbeing, where the relationship between the social capital components of ‘*social networks*’ and ‘*social trust*’ highly correlated with a sense of wellbeing. A strength of the study has, therefore, been to highlight real world exemplifications of how schools sought to build pupils’ personal capital by offering opportunities already available to advantaged children. Schools perceived that this process improved the potential for pupils to flourish through laying the foundations for improving their life chances and attainment as future individuals (4, 102). However, as little existing literature was found about the links between schools’ roles in promoting wellbeing and cultural and social capital, it appears that this area would benefit from further research.

### 4.4 Building on virtuous cycles

**Figure 7:**
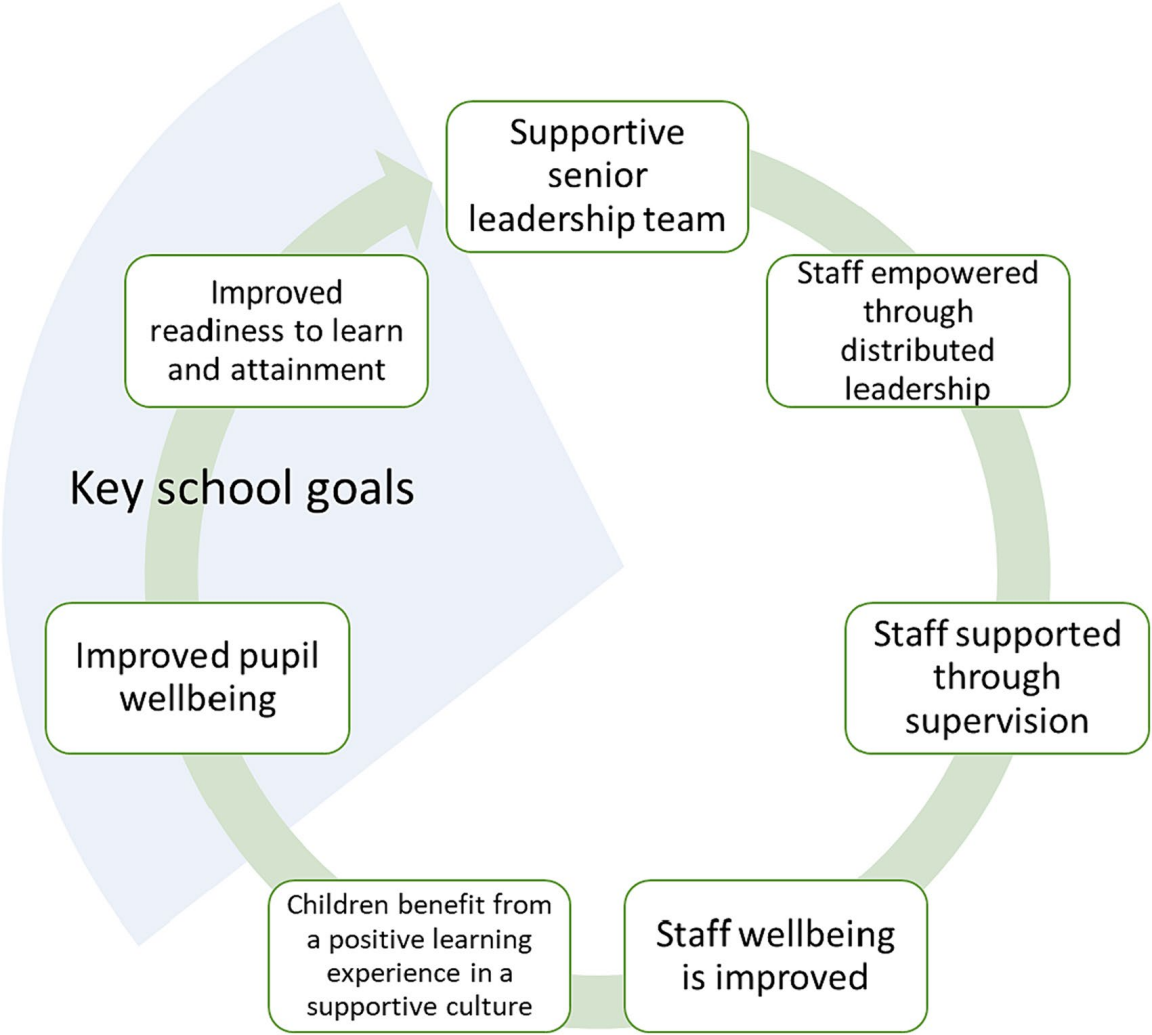
A virtuous cycle for wellbeing in the case study school.

The value of a dynamic and sustained approach to wellbeing promotion is recognized by the Department for Education (3) (p. 3) as ‘*a virtuous cycle*.’ Pulimeno et al. (103) argue that wellbeing promotion should be viewed as part of the virtuous cycle that enables pupils to attain academically and flourish, rather than as a standalone activity. It appears valuable to test Pulimeno et al.’s (103) argument, as the model may have potential to explain how wellbeing promotion links to key school goals, the importance of which has been established in principle two. Applying the virtuous cycle model to the case study school provided an alternative systems-based presentation of the components of wellbeing promotion as opposed to discrete factors (Figure 7).

The model highlights how Jane’s supportive leadership style was operationalized through a collaborative approach giving agency to staff, at the same time supporting them through providing supervision. The benefits were recognized by Tom and Jane to improve staff wellbeing which, in turn, provided positive role models for other staff and pupils and a positive learning environment for pupils.

This model appears suitable as a lens through which to understand the interrelationships between elements of whole-school wellbeing promotion. As this example considered only a fraction of the case study school’s wellbeing practice and the context in which it exists, it reveals that rather than a single virtuous cycle of wellbeing within a school, multiple virtuous circles exist concurrently (51). There may be merit in further research exploring the impact of interrelationships between the components and contexts of whole-school wellbeing promotion using the dataset from this study.

### 4.5 Managing complexity and context

Scholars argue that for schools to successfully enable all children to flourish they need to reduce uncertainty when implementing whole-school wellbeing promotion (104–106). Evidence demonstrates that high-quality implementation supports sustained embeddedness of school practices (27, 51). Where this fails, outcomes may not meet expectations, even though unrecognized complexities and contextual factors may be responsible for the underperformance (80, 105, 106). Management, therefore, involves an understanding of the effects of interacting contextual factors on the intervention (27).

At a group level, parents’ perceptions and concerns shaped wellbeing practice with Jane seeking to reduce parental anxiety amongst a sub-group of parents. Likewise, Clarke, Bunting and Barry (79) emphasized the influence of parents’ attitudes on promoting wellbeing. Thus, there is merit in exploring how uncertainty can be managed by schools to maximize the benefits of wellbeing practice for pupils (104–106). WHO advocates systems thinking as a framework to understand complexity, enabling organizations to *‘operat[e] more successfully and effectively in complex, realworld settings*’ (105) (p. 19). Systems thinking seeks to make sense of what is happening by looking holistically at the interdependencies of systems rather than focusing on component parts (104, 105). Rosas (107) advocates that a systems perspective is a useful lens for promoting wellbeing in school settings to understand complexity and dynamism.

Jane’s transformational leadership style which encouraged distributed leadership amongst teachers, echoes Kahn et al.’s (106) (p. 4) assertion that, from a systems-thinking approach, leadership needs to be ‘*action-based*,’ not ‘*role-based*.’ Jane, therefore, may be considered a ‘*complexity-inspired*’ leader who perceives the value of collaboration, communication and recognition that teachers are best placed to adapt content and pedagogy for their cohorts (106) (p. 5). This gives further weight to the value of framing wellbeing promotion using the virtuous cycle concept, understanding practice as a system of small positive steps. Its apparent usefulness for understanding whole-school wellbeing promotion in the case study school demonstrates its potential for schools more broadly.

Furthermore, the analysis highlighted the impact of macro level governmental policies on whole-school wellbeing promotion. Whilst education policy advocates pupil wellbeing as a key goal, the governmental focus of assessment remains on literacy and numeracy in primary schools. It might, therefore, be argued that a macro-level focus on how wellbeing promotion is evaluated has the potential to raise its perceived importance. This may result from investment in specially trained staff to make qualitative judgments about wellbeing, which could be used alongside existing local level evaluation tools such as self-evaluations and proxies for wellbeing (e.g., attendence statistics). In this study, insufficient funding manifested in a lack of staffing and other resourcing for wellbeing promotion and was highlighted by several schools. This lack of funding existed despite multiple government publications on the importance for schools to focus on the mental health and wellbeing of its pupils through whole-school initiatives (1, 3, 60, 92). This dichotomy raises the question of whether further funding is needed to improve the success of schools in enabling pupils to flourish.

### 4.6 Evaluating wellbeing promotion through different voices

Existing literature has called for better understanding about the benefits of whole school wellbeing promotion by listening to the views of children (84). A particular emphasis has focused on the different lived experiences between gender and socioeconomic status (SES) (17, 28, 36). This paper has highlighted the importance some schools placed on tailoring wellbeing practice to ‘*offset disadvantage*’ echoing previous literature highlighting the negative effect of disadvantage on wellbeing (108–110). Kennewell et al.’s (110) study of over 61,000 Australian children found that higher SES groups were more likely to engage in studying, music practice, youth organizations, sports, reading, chores, arts and crafts, and socializing with friends. These activities were more closely associated with flourishing.

The analysis highlighted how schools serving lower SES areas recognized the benefit of providing pupils with additional opportunities to develop skills, build experiences and promote cultural and social capital. In the case study school pupils reflected that residential and other school trips enabled periods of personal growth. Additionally, a sense of connection and belonging was important to the case study school’s vision, of particular benefit to children who may otherwise experiences a sense of isolation through perceived difference. The school recognized that facilitating these pupils to identify their ‘*tribes*’ promoted a beneficial sense of connection. This resonates with Dex and Hollingworth’s (111) DfE-funded publication, which argues that children in care are a group whose voices need to be heard to successfully address their wellbeing needs. In relation to pupils with SEN, whilst this study anonymized pupils’ responses making it unable to isolate one child’s own experiences, it highlights the suitability of creative research methods for this group of children. In all, this study has sought to respond to the calls for more analysis of whole-school wellbeing promotion by subgroups of children (17, 28, 36). However, it is recognized that was only partly met and further research with an appropriate research design would yield a richer understanding for shaping ongoing wellbeing practice (111).

### 4.7 Limitations

It is recognized that some limitations in undertaking this study exist. The research design was modified due to school closures during the COVID-19 pandemic resulting in changing the weighting from a project based on 70% primary data collection to 70% secondary data analysis. The revised design used a single English primary school as the case study and elicited the responses of 17 year six pupils. Thus, an intended participatory element with pupils involved as co-researchers was lost and there were fewer rich data about pupils’ lived experiences of whole-school wellbeing promotion. It is also recognized that the scope of this article does not provide schools with practical information such as funding requirements and other logistics which are required for implementation of wellbeing practice. These limitations may be addressed in future research.

### 4.8 Implications for professional practice and education policy

This paper presents a plethora of real-world examples of how schools promote pupils’ wellbeing. A purpose is to convince the reader that schools can undertake whole-school wellbeing promotion without ‘reinventing the wheel.’ It has emphasized how the sample schools used wellbeing practices and outcome measures that were already in existence, taking best practice from other school settings and adopting existing staff and pupil surveys to monitor outcomes. Schools took existing practice and adapted it to make it more relevant for their specific school setting. It is, therefore, hoped that approaches of English primary schools identified in this study are equally transferable to schools within the English education system and more broadly.

### 4.9 Conclusion

This study addressed remaining gaps in the substantial existing knowledge about whole-school wellbeing promotion by answering multiple sub-questions at each phase in the research design. This new knowledge was integrated into a final broader analysis which addressed the overall research question. This resulted in an original response to four areas where research remains scant: (1) initiatives within primary schools, (2) schools within the English education system, (3) the contextual factors which promote or hinder such initiatives and (4) pupils’ lived experiences of whole-school wellbeing promotion. Findings from 27 English primary schools in phases 2 and 3 of the study demonstrated how schools planned, implemented and assessed whole-school wellbeing promotion through formal and informal activities in the school setting, outdoors and through extracurricular opportunities. It highlighted how learning and school culture was adapted through values and practices which promoted wellbeing and that schools used formal assessment, self-evaluations and observations to evaluate school practice and pupil and staff perceptions of wellbeing. Additionally, the findings suggested that schools were challenged by contextuals factors at macro and local levels including schools funding levels, staffing and parental concerns. Despite the challenges of researching during the pandemic, the mapping activity elicited 17 year six pupils’ lived experiences in a school using a whole-school approach to wellbeing.

The study has contributed to this topic by presenting new knowledge as a set of six evidence-based principles in an attempt to make them of most value for researchers, teaching professionals and other interested readers. It recognized that wellbeing can be promoted by adapting existing practices and resources to the specific school setting as well as expanding current school activities to avoid ‘reinventing the wheel.’ At the same time, it also highlighted that schools were challenged by insufficient staffing and resourcing to adequately promote wellbeing and raises a question over the requirement for additional government funding to enable schools to deliver the whole-school initiatives it recommends.

This article demonstrates how schools enable pupils to flourish through taking a whole-school, salutogenic approach to wellbeing practice, develop social and cultural capital for wellbeing, sustain practice through building on multiple virtuous cycles of wellbeing, and manage complexity and context. It also highlighted how, by listening to pupils’ views, whole-school wellbeing promotion could be adapted for sub-groups of pupils. Whilst recognizing that further research is required, it highlighted that using creative methods such as the mapping activity provided an inclusive approach to capturing children’s voices alongside other stakeholders.

## Data Availability

The original contributions presented in the study are included in the article/supplementary material, further inquiries can be directed to the corresponding author.
